# Improved Sensitivity of Surface-Enhanced Raman Scattering with Gold Nanoparticles-Insulator-Metal Sandwich Layers on Flat Sapphire Substrate

**DOI:** 10.3390/nano11092416

**Published:** 2021-09-16

**Authors:** Wenbing Li, Xin Tong, Zhuo Yang, Jiali Zhang, Bo Liu, Chao Ping Chen

**Affiliations:** 1 Shanghai Industrial μTechnology Research Institute, Shanghai 201800, China; quentin.li@sitrigroup.com (W.L.); tongxin@shu.edu.cn (X.T.); yz98@shu.edu.cn (Z.Y.); zjl1201@shu.edu.cn (J.Z.); 2 School of Microelectronics, Shanghai University, Shanghai 201899, China; 3 Smart Display Lab, Department of Electronic Engineering, Shanghai Jiao Tong University, Shanghai 200240, China

**Keywords:** surface enhanced Raman scattering, gold nanoparticles, gold nanoparticles-insulator-metal sandwich layers, finite-difference time-domain method

## Abstract

Surface-enhanced Raman scattering (SERS) as a high sensitivity analytical method for molecule detection has attracted much attention in recent research. In this work, we demonstrated an improved SERS substrate, which has the gold nanoparticles randomly distributed on a SiO_2_ interception layer over a gold thin film layer on the flat sapphire substrate (AuNP/SiO_2_/Au/Sapphire), over the dispersed gold nanoparticles on a silicon substrate (AuNP/Si), for detection of R6G (1 × 10^−6^ M) in a Raman microscope. The fabrication of sandwich layers on top of the sapphire substrate involves evaporation of a gold mirror as thick as 100 nm, plasma enhanced chemical vapor deposition of the silica insulator layer 10 nm thick, and evaporation of a thin gold layer 10 nm thick for forming gold nanoparticles. For comparison, a gold thin film with a thickness of 5 nm and 10 nm was evaporated on a silicon substrate, respectively (AuNP/Si), as the reference SERS substrates in the experiment. The AuNP/SiO_2_/Au/Sapphire substrate demonstrated improved sensitivity in detection of molecules in Raman microscopy, which can enable the molecules to be recognizable at a low laser power as 8.5 × 10^−3^ mW, 0.017 mW, 0.085 mW, and 0.17 mW for ultrashort exposure time. The simulation of AuNP/SiO_2_/Au/Sapphire substrate and AuNP/Si substrate, based on the finite-difference time-domain (FDTD) method, explained the improved sensitivity for detection of R6G molecules from the view of classical electromagnetics, and it suggested the optimized size for the gold nanoparticles and the optimized laser wavelength for Raman microscopy for further research.

## 1. Introduction 

Surface-enhanced Raman scattering (SERS) has attracted interest for its high sensitivity in detection of molecules [1,2,3] since its discovery in 1974, in the testing of pyridine adsorbed on the surface of a silver electrode [4,5]. It is widely accepted that the rough gold (silver) thin film [6,7,8], gold (silver) nanoparticles [9,10,11,12,13,14,15], and nanopatterned gold (silver) thin film [16,17,18,19,20], are the keys for enhancing the signatures of molecules in the Raman microscopy, since there are highly localized electric fields, namely, the “hotspots” formed close to these nanoscale metallic structures upon the incidence of light [21,22,23]. The molecules adsorbed on the high density “hotspots” contribute most of the enhancement for the surface enhanced Raman microscopy [22,24].

The chemical synthesized gold or silver colloids that are used as the substrate for enhancing the Raman scattering for detection of dyes started at least as early as the 1980s [13]. It was even reported to achieve single molecule level detection [25]. However, the production of the gold and silver colloids involves the use of hazard aqueous chemicals, such as HAuCl_4_ and erosive acids [26], and it may give rise to the risk of causing health and environmental problems [27,28]. The gold nanoparticles produced by evaporation and thermal anneal need neither any complex chemical process or hazard aqueous solutions, nor expensive facilities for pattern transfer, such as electron beam lithography [29]. Furthermore, it also has the advantage of producing uniform gold nanoparticles on a wafer scale, rather than an area as small as a few square millimeters through high resolution lithography [9]. Combined with a gold mirror, the gold nanoparticles over a gold thin film intercepted by a thin dielectric layer [9,30], or the gold nano dimers on the gold mirror [31], reached an even higher sensitivity in SERS.

In this work, we presented gold nanoparticles-insulator-metal sandwich layers on the flat sapphire substrate (AuNP/SiO_2_/Au/Sapphire) to improve the sensitivity of SERS. It was combined of a flat sapphire substrate, a metal mirror made of a gold thin film with a thickness of 100 nm, a SiO_2_ dielectric thin film with a thickness of 10 nm, and randomly distributed gold nanoparticles produced from a 10 nm thick gold thin film, sequentially. For a comparison study, the samples with randomly distributed gold nanoparticles on a silicon substrate (AuNP/Si) were also used in the Raman microscopy of R6G molecules with a concentration of 1 × 10^−6^ M. Our work presented that the AuNP/SiO_2_/Au/Sapphire substrate improved the sensitivity of SERS for the detection of R6G more than the AuNP/Si substrate. Meanwhile, the simulation based on FDTD method uncovered the physics behind the experiment, and it concluded that the reason for the improvement was from a higher enhancement of the scattered field with the AuNP/SiO_2_/Au/Sapphire substrate, rather than randomly distributed gold nanoparticles on a silicon substrate.

## 2. Materials and Methods

### 2.1. Preparation of Materials

The gold mirror as thick as 100 nm was evaporated on the surface of a 2-inch sapphire substrate with an adhesion layer of Cr as 2 nm at first, and then a 10 nm thick SiO_2_ layer was deposited on the surface of the gold mirror by plasma enhanced chemical vapor deposition (PECVD). The second layer of gold thin film with a thickness of 10 nm was evaporated on the surface of the SiO_2_ thin layer. The gold nanoparticles were formed through a thermal process at 600 deg C for 30 min in a tube furnace. For comparison, two other silicon substrates were evaporated of a gold thin film as 5 nm and 10 nm respectively, and the gold nanoparticles were formed through thermal anneal in the tube furnace at the same condition. 

The target detection molecule R6G (1 × 10^−6^ M) was prepared by dilution of R6G powder in deionized water with a flask. After stabilization of the R6G solution, a small amount of the solution was moved to a petri dish. The AuNP/SiO_2_/Au/Sapphire substrate and AuNP/Si substrate were moved to the petri dish and immersed in the R6G (1 × 10^−6^ M) solution for a whole night to allow the R6G molecules to adsorb on the surface.

### 2.2. Characterization Methods

The three types of substrates, gold nanoparticles formed by 5 nm gold thin film on silicon substrate (AuNP/Si), gold nanoparticles formed by 10 nm gold thin film on silicon substrate (AuNP/Si), and AuNP/SiO_2_/Au/Sapphire substrate were symbolized as sample S1, sample S2, and sample S3, respectively, after their preparation. The surface morphology of the gold nanoparticles on each substrate was first inspected by an atomic force microscope (Bruker Dimension Icon, Bruker Corporation, Billerica, MA, USA). For inspection of the layer structure of sample S3 (AuNP/SiO_2_/Au/Sapphire), a small piece was cut from the substrate for milling a tiny trench on its surface with a focused ion beam miller (FEI Helios NanoLab 600 Dual Beam FIB SEM, Thermal Fisher Scientific, Waltham, MA, USA) to expose its cross section. The gold nanoparticle distribution on the surface of sample S3, and the tiny trench on its surface, were inspected by a scanning electron microscope (FEI Helios NanoLab 600 Dual Beam FIB SEM, Thermal Fisher Scientific, Waltham, MA, USA) in the same facility. Samples S1, S2, and S3 were immersed in the R6G (1 × 10^−6^ M) as prepared, and they were dried for the Raman microscopy (Renishaw Invia, Renishaw Corporation, Wotton-under-Edge, England) for inspection with an incident laser wave set at 633 nm, at various injection laser powers and exposure times. A 50X objective was used in the Raman measurement.

### 2.3. Calculation Based on Electromagnetics Theory

The computational electromagnetics theory was widely used to understand the physics of plasmonics in various experiment works [32,33,34]. In order to understand the enhancement of the Raman signatures by gold nanoparticles-based substrates, simulations based on the finite-difference time-domain method were used to explore the physics behind the phenomena. It was a numerical method used to discrete the Faraday equation and Ampere equation from Maxwell’s equations group in the three-dimensional space and time domain, and therefore the electric field and magnetic field at each Yee grid and time step could be calculated with predefined injection light source [35]. The finite spatial grid, named Yee cell, was connected with the finite time step with the Courant stability condition to make sure the light velocity was a constant while transporting in various media. The perfectly matched layers (PMLs) were defined to enclose the simulation object and injection light source with a distance of one wavelength from the simulation object, and therefore the light scattered by the simulation object could be absorbed fully at the boundary with matched impedance [36]. In this work, the calculation was implemented with FDTD solutions.

In our simulation model, the gold nanoparticle was assumed as a perfect nanosphere for simplification of the calculation. The calculation was implemented on a single sphere gold nanoparticle on a silicon substrate (AuNP/Si) and a single sphere gold nanoparticle on a sapphire substrate with a silicon dioxide thin film (10 nm) as a spacer layer over a gold mirror (AuNP/SiO_2_/Au/Sapphire) at first. A total field scattered field (TFSF) source, with a broad wavelength range that covered the visible region from 400 nm to 800 nm, was used as the incident light source, and the PMLs were used as the absorption boundary for absorbing the scattered waves outside the simulation area. The *n* and *k* values of gold, silicon dioxide, silicon, and sapphire were from references [37,38], and they were fitted with the FDTD fitting tool with a RMS error less than or close to 0.1 before the running of the simulation models. The scattered cross section was defined as the ratio between the scattered power (with the unit as watt) over the injection light intensity (with the unit as W/m^2^) in the simulation region. The enhancement factor of Raman scattering was calculated as the fourth order of the ratio between the electric field (with a unit as V/m) of the scattered light and the electric field (with a unit as V/m) of the incident laser wavelength [39]. According to Yang’s research [22], the hotspots that had the largest enhancement factor contributed most to the magnification of the Raman signatures, the maximum enhancement factor that in a tiny cubic region that encloses the simulation object (single or multiple sphere gold nanoparticles) was calculated to evaluate the model for enhancing the Raman signatures of target molecules that adsorbed to the surface of corresponding SERS substrates. A further simulation, that considered the distribution of the gold nanoparticles and the distribution function extracted out from a tiny region of the SEM image of sample S3, was used to generate the randomly distributed gold nanoparticles for the model AuNP/Si and the model AuNP/SiO_2_/Au/Sapphire. A random function, that was assigned by the standard deviation and mean diameter of gold nanoparticles extracted out from a tiny region of the SEM image, was used to generate the size of each gold nanoparticle in the simulation region. An amount of 30 simulations for each model, with different distributions of gold nanoparticles for each running, were implemented to calculate the maximum enhancement factor for a comparison of the model AuNP/Si and the model AuNP/SiO_2_/Au/Sapphire.

## 3. Results and Discussion

### 3.1. Surface Morphology of AuNP/Si and AuNP/SiO_2_/Au/Sapphire Substrates

**Figure 1:**
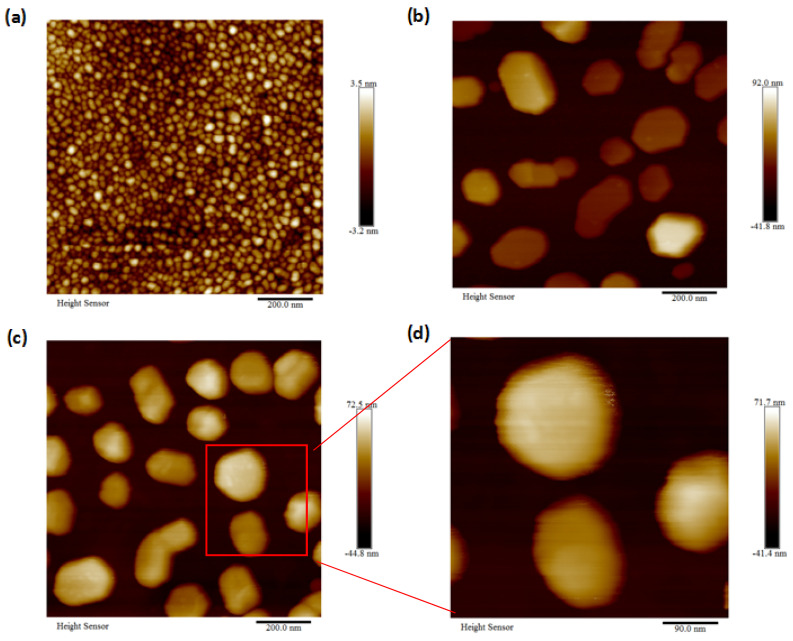
AFM images of the gold nanoparticles on samples S1, S2, and S3. (**a**) Gold nanoparticles on the surface of sample S1. (**b**) Gold nanoparticles on the surface of sample S2 (**c**,**d**) Gold nanoparticles on the surface of sample S3.

**Figure 2:**
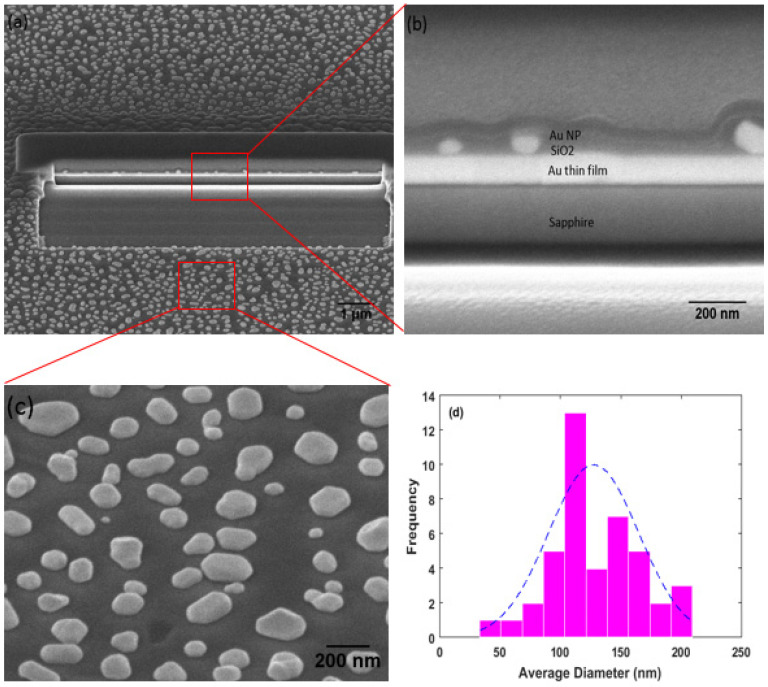
The SEM images of sample S3 and the gold nanoparticles distribution on the surface of S3. (**a**) Cross section of S3 after the focus ion beam cutting. (**b**) Cross section of S3 showed the gold nanoparticles-SiO_2_-gold thin film sandwich layers structure. (**c**) Gold nanoparticles on the surface of SiO_2_. (**d**) Distribution of the gold nanoparticles on surface of S3 from SEM image.

**Figure 3:**
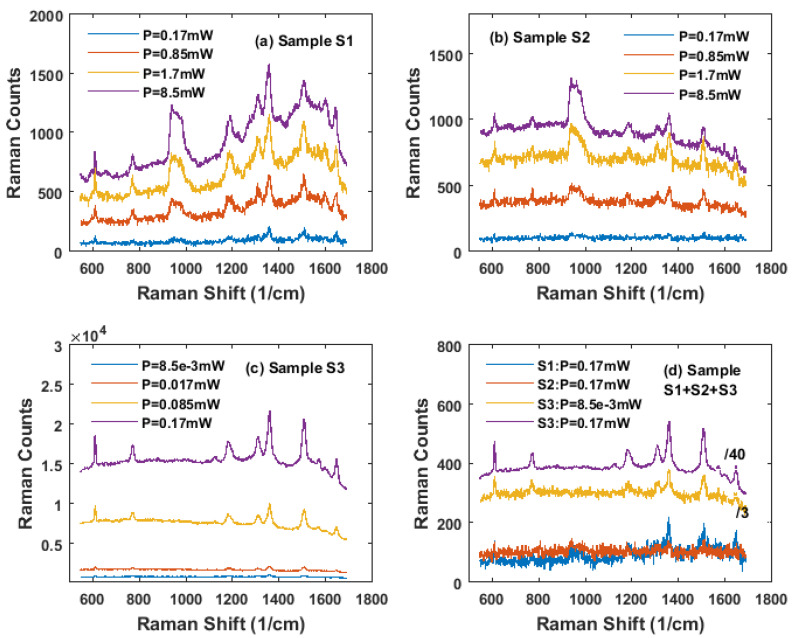
SERS measurement of R6G with a concentration of 1 × 10^−6^ M with different samples. The laser excitation was 633 nm, exposure time was 5 s for all measurements in (**a**–**d**), and the objective was 50X. For (**a**,**b**), the measurement taken at different laser powers of 0.17 mW, 0.85 mW, 1.7 mW, and 8.5 mW. (**a**) Sample S1 5 nm evaporated gold thin film on silicon substrate annealed at 600 C for 30 min. (AuNP/Si) (**b**) Sample S2 10 nm evaporated gold thin film on silicon substrate annealed at 600 C for 30 min (AuNP/Si). (**c**) Sample S3 10 nm evaporated gold thin film on SiO_2_ (10 nm) Au (100 nm) Cr (2 nm) on flat sapphire substrate annealed at 600 C for 30 min (AuNP/SiO_2_/Au/Sapphire). The measurement taken at the different laser powers of 8.5 × 10^−3^ mW, 0.017 mW, 0.085 mW, and 0.17 mW. (**d**) Comparison of Samples S1, S2, and S3 at different laser power in the test. Sample S1 and S2 at *p* = 0.17 mW, S3 at *p* = 0.17 mW and *p* = 8.5 × 10^−3^ mW. The curve for sample S3 at the laser power of 0.17 mW and 8.5 × 10^−3^ mW was divided by 40 and 3, respectively, for combination of the four curves in the same diagram.

**Figure 4:**
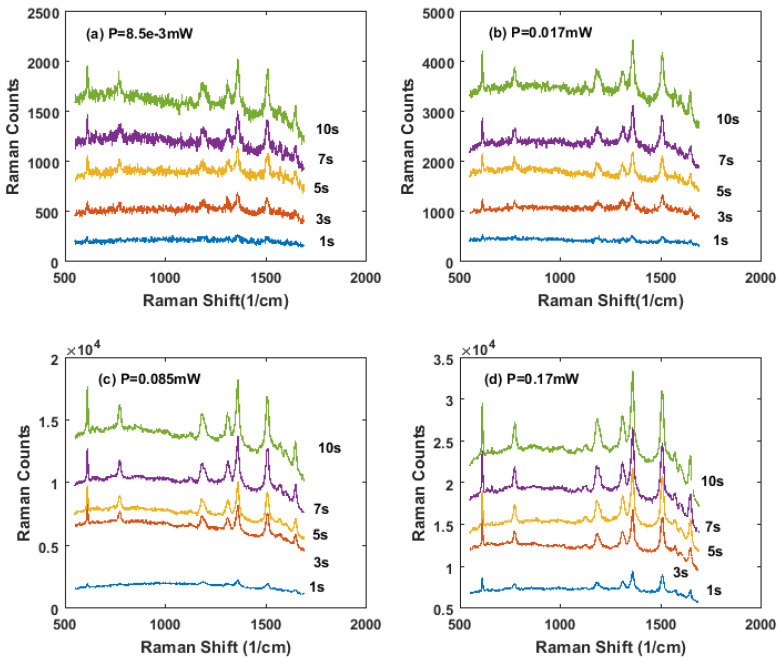
SERS measurement of R6G = 1 × 10^−6^ M with sample S3 at different laser powers and different exposure times. The laser excitation for all (**a**–**d**) were 633 nm, and objective was 50X. Different exposure times at 1 s, 3 s, 5 s, 7 s, and 10 s were set for each test. (**a**) The laser power was 8.5 × 10^−3^ mW; (**b**) the laser power was 0.017 mW; (**c**) the laser power was 0.085 mW; (**d**) the laser power was 0.17 mW.

**Figure 5:**
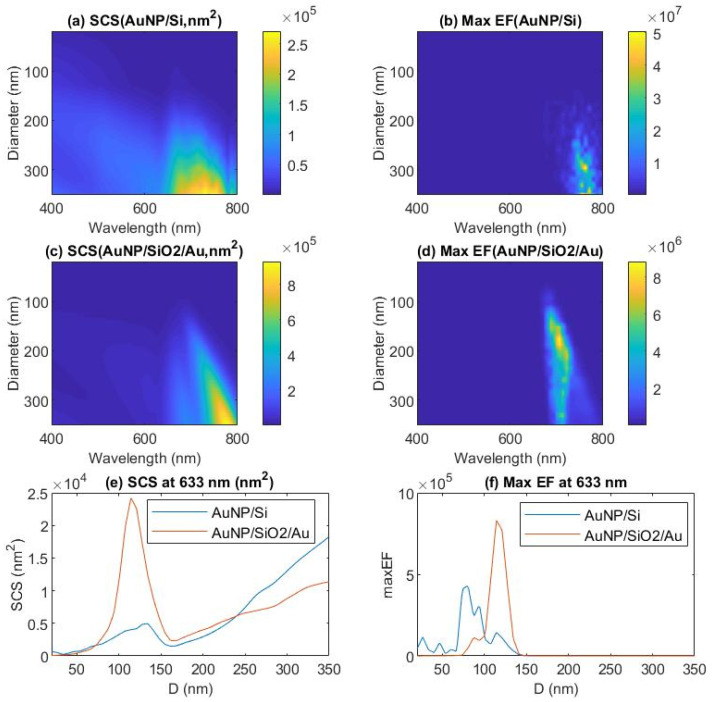
FDTD simulation of single sphere gold nanoparticle on a silicon substrate (AuNP/Si) and single sphere gold nanoparticle on silicon dioxide interception layer (10 nm) over a gold mirror (100 nm) on a sapphire substrate (AuNP/SiO_2_/Au/Sapphire). (**a**) Scattering cross section of a single sphere gold nanoparticle for model (AuNP/Si) depends on sphere diameter and incident wavelength. (**b**) Maximum enhancement factor (/E/\/E_0_/)^4^ of a single sphere gold nanoparticle for model (AuNP/Si) depends on sphere diameter and incident wavelength. (**c**) Scattering cross section of a single sphere gold nanoparticle for model (AuNP/SiO_2_/Au/Sapphire) depends on sphere diameter and incident wavelength. (**d**) Maximum enhancement factor (/E/\/E_0_/)^4^ of a single sphere gold nanoparticle for model (AuNP/SiO_2_/Au/Sapphire) depends on sphere diameter and incident wavelength. (**e**) Scattering cross section of a single sphere gold nanoparticle for model (AuNP/Si) and model (AuNP/SiO_2_/Au/Sapphire) depends on sphere diameter at an incident wavelength of 633 nm. (**f**) Maximum enhancement factor (/E/\/E_0_/)^4^ of a single sphere gold nanoparticle for model AuNP/Si and model AuNP/SiO_2_/Au/Sapphire depends on sphere diameter at an incident wavelength of 633 nm.

**Figure 6:**
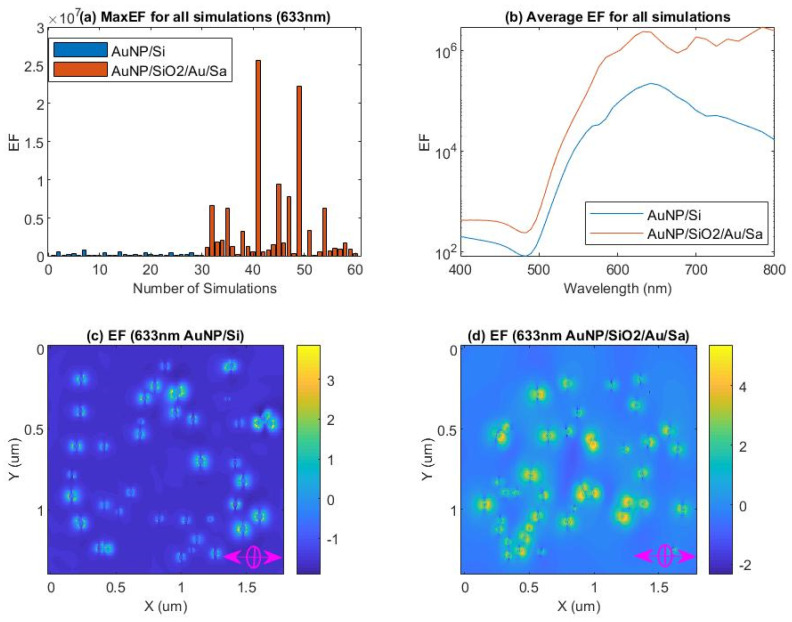
FDTD simulation of randomly distributed gold nanoparticles on silicon substrate (AuNP/Si) and randomly distributed gold nanoparticles on a silicon dioxide interception layer over a gold mirror on sapphire substrate (AuNP/SiO_2_/Au/Sapphire). (**a**) Maximum enhancement factor (\E\/\E_0_\)^4^ at incident wavelength 633 nm for each run of model AuNP/Si and model AuNP/SiO_2_/Au/sapphire over an amount of 30 simulations, respectively. (**b**) Average value of the maximum enhancement factor (\E\/\E_0_\)^4^ depends on the incident wavelength over an amount of 30 simulations for model AuNP/Si and model AuNP/SiO_2_/Au/Sapphire, respectively. (**c**) Randomly selected enhancement factor (\E\/\E_0_\)^4^ at the foot of randomly distributed gold nanoparticles on the surface of a silicon substrate (at XY plane) for model AuNP/Si with an incident wavelength of 633 nm. The color bar of the enhancement factor was in log scale (log{ (\E\/\E_0_\)^4^}). (**d**) Randomly selected enhancement factor (\E\/\E_0_\)^4^ image at the foot of randomly distributed gold nanoparticles on the surface of the SiO_2_ interception layer (at XY plane) for model AuNP/SiO_2_/Au/Sapphire with an incident wavelength of 633 nm. The color bar of the enhancement factor was in log scale (log{(\E\/\E_0_\)^4^}).

The surface morphology of the gold nanoparticles on the three samples characterized by AFM was presented in Figure 1a–d. The SEM images with an angled view of the surface and cross section of sample S3 were shown in Figure 2a–d. The Raman microscopy of R6G molecules on each substrate with an excitation laser wave as 633 nm was displayed in Figure 3 and Figure 4. The simulation result, based on FDTD method for a perfect single gold nanoparticle and randomly distributed gold nanoparticles for each model (AuNP/Si and AuNP/SiO_2_/Au/Sapphire), was displayed in Figure 5 and Figure 6, respectively. 

From the AFM images shown in Figure 1, the sizes of the gold nanoparticles for sample S2 and S3 were similar to each other, with a color bar scale the third dimension among (−41.8 nm, 92 nm) and (−44.8 nm, 72.5 nm), respectively. For sample S1, the size of the gold nanoparticles was much smaller than that of samples S2 and S3. It had an average diameter of about 23 nm. The difference was from the thickness of the gold thin film, in which the gold thin film that was evaporated on S1 was 5 nm and the gold thin film that was evaporated on S2 and S3 was 10 nm, respectively. The cross section of the gold nanoparticles-insulator-metal sandwich layers was shown in Figure 2a at a tilt view of 45° in the SEM, and a magnified SEM image was shown in Figure 2b. The layer structure of gold nanoparticles on the SiO_2_ interception layer over the gold mirror was quite clear. Another magnified SEM image of the surface of the gold nanoparticles on sample S3 was shown in Figure 2c, also with a tilt view of 45°. The distribution of the diameter for the gold nanoparticles in Figure 2c was given in Figure 2d, which gave an average diameter of about 129 nm with a standard deviation of about 38 in that area (mu = 128.581, sigma = 37.9788).

### 3.2. Raman Microscopy of R6G Molecules Adsorbed on AuNP/Si and AuNP/SiO_2_/Au/Sapphire Substrates

In Figure 3, the diagrams presented the Raman scattering curves of R6G taken at different laser powers of 0.17 mW, 0.85 mW, 1.7 mW, and 8.5 mW for samples S1 and S2 (Figure 3a,b). The Raman signatures of R6G on sample S3 taken at different laser powers of 8.5 × 10^−3^ mW, 0.017 mW, 0.085 mW, and 0.17 mW shown in Figure 3c was much lower than that for sample S1 and sample S2. For all three samples in this test, the exposure time was 5 s, and the only difference was the laser power. All the signatures of R6G at 610 cm^−1^, 770 cm^−1^, 1124 cm^−1^, 1180 cm^−1^, 1309 cm^−1^, 1361 cm^−1^, 1502 cm^−1^, 1535 cm^−1^, 1572 cm^−1^, 1599 cm^−1^, and 1649 cm^−1^, together with the signature from silicon between 939 cm^−1^ and 980 cm^−1^, were clear for sample S1, taken at the laser powers of 0.85 mW, 1.7 mW, and 8.5 mW. When the laser power decreased to 0.17 mW, only a few main signatures of 610 cm^−1^, 770 cm^−1^, 1180 cm^−1^, 1361 cm^−1^, 1502 cm^−1^, and 1649 cm^−1^ can be recognized, while other signatures were lost in the noise. For sample S2, all the signatures for R6G were clear when taken at the laser powers of 0.85 mW, 1.7 mW, and 8.5 mW, respectively, but for the curves taken at the laser power of 0.17 mW, all the signatures for R6G were lost in the noise, in which they became difficult to distinguish. However, for the measurement of sample S3, the laser power was decreased to 8.5 × 10^−3^ mW, 0.017 mW, 0.085 mW, and 0.17 mW, respectively, in which the biggest laser power was equal to the smallest laser power in the test of sample S1 and S2. However, all the signatures of the R6G could still be recognized for the tests taken at the laser powers 8.5 × 10^−3^ mW, 0.017 mW, 0.085 mW, and 0.17 mW. Compared with sample S1 and sample S2, the noise in the diagram for sample S3 was much lower. For a further comparison, the Raman curves for all three samples S1, S2, and S3 taken at the laser power of 0.17 mW were put in the same diagram in Figure 3d, and the Raman curve for sample S3 taken at the lowest laser power 8.5 × 10^−3^ mW was added in the same diagram. The Raman counts for sample S3 taken at 0.17 mW and 8.5 × 10^−3^ mW were divided by 40 and 3, respectively, in Figure 3d for using a smaller scale to show the curves for sample S1 and sample S2 clearly. The signatures of R6G for sample S3 at 0.17 mW and 8.5 × 10^−3^ mW were clear and easy to recognize, while the signatures of R6G for sample S1 (only a few main signatures can be recognizable) and sample S2 were difficult to distinguish in the noise. The result indicated that, with the help of the gold mirror, the sensitivity of the S3 in SERS was much improved.

Further study of sample S3 taken at different exposure times at 1 s, 3 s, 5 s, 7 s, and 10 s, for each laser power, was displayed in Figure 4. It was obvious that for Raman curves taken at laser power 0.085 mW and 0.17 mW for all different exposure times, from 1 s to 10 s, the signatures of R6G were easy to distinguish. For the one taken at laser power 0.017 mW, the Raman signatures of R6G were still recognizable at 1 s. For the lowest laser power 8.5 × 10^−^^3^ mW, the R6G signatures were obvious for exposure times of 3 s, 5 s, 7 s, and 10 s. Even when the exposure time decreased to 1 s, some main signatures of R6G were still recognizable.

### 3.3. FDTD Simulation Results for Modeling AuNP/Si and AuNP/SiO_2_/Au/Sapphire

The simulation result of a perfect single sphere gold nanoparticle for model AuNP/Si and AuNP/SiO_2_/Au/Sapphire was displayed in Figure 5. The scattered cross section for AuNP/Si and AuNP/SiO_2_/Au/Sapphire depended on the incident wavelength and the diameter of the gold nanosphere, as shown in Figure 5a,c, respectively. The two models showed a similar trend of the variation of the scattered cross section, depending on nanosphere diameter and incident wavelength, but the detail distribution of the scattered cross section was different to each other. It was observed that the gold nanospheres with smaller diameters (almost under 100 nm) showed a smaller scattered cross section that was lower than 1 × 10^4^ nm^2^ over the whole visible range. With the increase of the diameter, the scattered cross section improved much, in that a large area in the image owned the scattered cross section value approached or above 1 × 10^4^ nm^2^. The maximum value of the scattered cross section at around 1 × 10^5^ nm^2^ was achieved in a small region with a larger diameter and longer wavelength. The scattered cross section curve at incident wavelength 633 nm for the model AuNP/Si and AuNP/SiO_2_/Au/Sapphire was extracted out from Figure 5a,c, and displayed in Figure 5e. The curve for the model AuNP/SiO_2_/Au/Sapphire showed a larger scattered cross section over the model AuNP/Si among 100 nm to 150 nm, which means that more light power was scattered by the gold sphere nanoparticle, and it therefore contributed to the enhancement of Raman signatures, rather than being absorbed by the metallic nanostructure on the substrate. The maximum enhancement factor of the scattered field depended on the diameter of the gold sphere nanoparticle, and the incident wavelength for the model AuNP/Si and model AuNP/SiO_2_/Au/Sapphire was displayed in Figure 5b,d, respectively. The highest maximum enhancement factor for the model AuNP/Si happened in a small region that, with a long incident wavelength and a big diameter, covered the diameter range from 150 nm to 350 nm and the incident wavelength range from 680 nm to 800 nm. By contrast, for model AuNP/SiO_2_/Au/Sapphire, the highest maximum enhancement factor was located in a triangle region in the image that covered the diameter range from 75 nm to 350 nm and the incident wavelength from 700 nm to 800 nm. The maximum enhancement factor value at an incident wavelength of 633 nm was extracted from Figure 5b,d and displayed in Figure 5f. It was evident that the maximum enhancement factor for model AuNP/SiO_2_/Au/Sapphire (8.3 × 10^5^) was about 5.7 times of that for model AuNP/Si (1.444 × 10^5^) in case the diameter was 114 nm. In the diameter range between 99 nm and 139 nm, the maximum enhancement factor for model AuNP/SiO2/Au/Sapphire was higher than that of model AuNP/Si. The peak value of the maximum enhancement factor 4.238 × 10^5^ for model AuNP/Si at an incident wavelength of 633 nm appeared at a diameter of 78 nm, and it quickly decreased one order in the case of the diameter smaller than 66 nm or larger than 139 nm. The simulation result of models AuNP/Si and AuNP/SiO_2_/Au/Sapphire can support the performance of sample S1, S2, and S3 in Raman microscopy.

For a calculation to be closer to practical distribution of the gold nanoparticles on a silicon substrate and on the SiO_2_ spacer over an Au mirror sitting on a sapphire substrate, a further simulation used the Gaussian function (mu = 128.581 nm, sigma = 37.9788) extracted out from Figure 2c,d to describe the distribution of gold nanoparticles for model AuNP/Si and model AuNP/SiO_2_/Au/Sapphire in a small area of 1.6 µm×1.2µm. The PML boundaries of six planes vertical to the X, Y, and Z axis, respectively, were kept at a distance of one wavelength from the edge of the simulation objects. An amount of 30 simulations for both model AuNP/Si and model AuNP/SiO_2_/Au/Sapphire were implemented, and for each simulation, the distribution of the gold nanoparticles was generated randomly with the same statistic function, where the result was displayed in Figure 6. The bar diagram in Figure 6a presented the maximum enhancement factor for each simulation, corresponding to model AuNP/Si and model AuNP/SiO_2_/Au/Sapphire with an incident wavelength of 633 nm, while the average value of the maximum enhancement factor, that dependent on the incident wavelength for model AuNP/Si and model AuNP/SiO_2_/Au/Sapphire, was displayed in Figure 6b. Though the maximum enhancement factor at an incident laser wavelength of 633 nm for each simulation of model AuNP/Si and AuNP/SiO_2_/Au/Sapphire was quite different to each other for a randomly generated distribution of gold nanoparticles, over a large amount of the simulations implemented for both models, model AuNP/SiO_2_/Au/Sapphire showed a higher maximum enhancement factor than that of model AuNP/Si from Figure 6a. It was obvious in Figure 6b that model AuNP/SiO_2_/Au/Sapphire had an advantage on the enhancement of the scattered field over model AuNP/Si among the visible range between 400 nm and 800 nm. Additionally, model AuNP/SiO_2_/Au/Sapphire (the average maximum enhancement factor was 2.37 × 10^6^ at 633 nm) had an average maximum enhancement factor that was 12 times over that of model AuNP/Si (the average maximum enhancement factor was 1.968 × 10^5^ at 633 nm) at an incident wavelength of 633 nm, which supported the test result with the Raman microscopy in Figure 3. Further, a randomly selected enhancement factor (\E/E0\^4^) image at the XY plane located at the foot of randomly distributed gold nanoparticles with an incident wavelength of 633 nm among the 30 simulations for model AuNP/Si and model AuNP/SiO_2_/Au/Sapphire was displayed in Figure 6c,d respectively. The incident light was injected toward the substrate (-Z axis) that was vertical to the XY plane, with a polarization direction parallel to the X axis. The color bar in Figure 6c, d was in log scale. From both images, the higher enhancement factor of the scattered field happened at the points that were close to the gold nanoparticles (or named as “hotspots”), and it decayed rapidly with the distance from the gold nanoparticles, thus a high density of gold nanoparticles, especially with more nanoparticles at an optimized size and illuminated under optimized incident laser wave, was critical for a higher enhancement of the scattered field.

## 4. Conclusions

We demonstrated that the gold nanoparticles-insulator-metal sandwich layers structure on the sapphire substrate had the advantage over the gold nanoparticles on the surface of silicon substrate for high sensitivity detection of R6G molecules in Raman microscopy, experimentally. The combination of the local surface plasmonic resonance from the gold nanoparticles and the surface Plasmon Polariton from the gold mirror led to further enhancement of the signatures from the molecules, thus resulting in an improved sensitivity in detection of molecules. Our simulation work based on the FDTD method calculated the scattered cross section, the maximum enhancement factor of a single nanosphere gold nanoparticle on a silicon substrate (AuNP/Si), and a single nanosphere gold nanoparticle on a silicon dioxide spacer (10 nm) over a gold mirror on a sapphire substrate (AuNP/SiO_2_/Au/Sapphire), and the result showed that model AuNP/SiO_2_/Au/Sapphire had an advantage on enhancing the scattered field more than AuNP/Si at an incident wavelength of 633 nm. Considering the influence of the distribution of gold nanoparticles, the Gaussian distribution extracted from an SEM image was used to generate the randomly distributed gold nanoparticles, and it implemented the simulation over 30 times for model AuNP/Si and model AuNP/SiO_2_/Au/Sapphire, respectively. In each simulation, the distribution of the gold nanoparticles was different from the others, but they followed the same statistic law. The result showed that model AuNP/SiO_2_/Au/Sapphire was better for magnification of the scattered field than model AuNP/Si among the visible region (400~800 nm), with a higher average maximum enhancement factor. With an incident wavelength of 633 nm that was used in the Raman microscopy in this experiment, the model AuNP/SiO_2_/Au/Sapphire showed an average maximum enhancement factor that was 12 times over the model AuNP/Si, which supported our experiments for the Raman microscopy of R6G on corresponding substrates.

## References

[1] Liu Z., Yang Z., Peng B., Cao C., Zhang C., You H., Xiong Q., Li Z., Fang J. (2014). Highly Sensitive, Uniform, and Reproducible Surface-Enhanced Raman Spectroscopy from Hollow Au-Ag Alloy Nanourchins. Adv. Mater..

[2] Yu X., Tao J., Shen Y., Liang G., Liu T., Zhang Y., Wang Q.J. (2014). A metal–dielectric–graphene sandwich for surface enhanced Raman spectroscopy. Nanoscale.

[3] Fang J., Du S., Lebedkin S., Li Z., Kruk R., Kappes M., Hahn H. (2010). Gold Mesostructures with Tailored Surface Topography and Their Self-Assembly Arrays for Surface-Enhanced Raman Spectroscopy. Nano Lett..

[4] Fleischmann M., Hendra P.J., McQuillan A.J. (1974). Raman spectra of pyridine adsorbed at a silver electrode. Chem. Phys. Lett..

[5] McQuillan A.J. (2009). The discovery of surface-enhanced Raman scattering. Notes Rec. R. Soc. J. Hist. Sci..

[6] Greeneltch N.G., Blaber M.G., Henry A.-I., Schatz G.C., Van Duyne R.P. (2013). Immobilized Nanorod Assemblies: Fabrication and Understanding of Large Area Surface-Enhanced Raman Spectroscopy Substrates. Anal. Chem..

[7] Lee C., Robertson C.S., Nguyen A.H., Kahraman M., Wachsmann-Hogiu S. (2015). Thickness of a metallic film, in addition to its roughness, plays a significant role in SERS activity. Sci. Rep..

[8] Kreno L.E., Greeneltch N.G., Farha O.K., Hupp J.T., Van Duyne R.P. (2014). SERS of molecules that do not adsorb on Ag surfaces: A metal–organic framework-based functionalization strategy. Analyst.

[9] Wang D., Zhu W., Best M.D., Camden J.P., Crozier K.B. (2013). Wafer-scale metasurface for total power absorption, local field enhancement and single molecule Raman spectroscopy. Sci. Rep..

[10] Ma Q., Zhang H., Liu W., Ge J., Wu J., Wang S., Wang P. (2016). Surface-enhanced Raman scattering substrate based on cysteamine-modified gold nanoparticle aggregation for highly sensitive pentachlorophenol detection. RSC Adv..

[11] Mevold A.H.H., Hsu W.-W., Hardiansyah A., Huang L.-Y., Yang M.-C., Liu T.-Y., Chan T.-Y., Wang K.-S., Su Y.-A., Jeng R.-J. (2015). Fabrication of Gold Nanoparticles/Graphene-PDDA Nanohybrids for Bio-detection by SERS Nanotechnology. Nanoscale Res. Lett..

[12] Jin Z.-M., Gu W., Shi X.-B., Wang Z.-K., Jiang Z.-Q., Liao L.-S. (2014). A Novel Route to Surface-Enhanced Raman Scattering: Ag Nanoparticles Embedded in the Nanogaps of a Ag Substrate. Adv. Opt. Mater..

[13] Lee P.C., Meisel D. (1982). Adsorption and surface-enhanced Raman of dyes on silver and gold sols. J. Phys. Chem..

[14] Xu W., Ling X., Xiao J., Dresselhaus M.S., Kong J., Xu H., Liu Z., Zhang J. (2012). Surface enhanced Raman spectroscopy on a flat graphene surface. Proc. Natl. Acad. Sci. USA.

[15] Liang H., Li Z., Wang W., Wu Y., Xu H. (2009). Highly Surface-roughened “Flower-like” Silver Nanoparticles for Extremely Sensitive Substrates of Surface-enhanced Raman Scattering. Adv. Mater..

[16] Wang N., Zhu W., Chu Y., Crozier K.B. (2012). High Directivity Optical Antenna Substrates for Surface Enhanced Raman Scattering. Adv. Mater..

[17] Wang D., Zhu W., Best M.D., Camden J., Crozier K. (2013). Directional Raman Scattering from Single Molecules in the Feed Gaps of Optical Antennas. Nano Lett..

[18] Kanipe K.N., Chidester P.P.F., Stucky G.D., Moskovits M. (2016). Large Format Surface-Enhanced Raman Spectroscopy Substrate Optimized for Enhancement and Uniformity. ACS Nano.

[19] Gillibert R., Sarkar M., Bryche J.-F., Yasukuni R., Moreau J., Besbes M., Barbillon G., Bartenlian B., Canva M., De La Chapelle M.L. (2016). Directional surface enhanced Raman scattering on gold nano-gratings. Nanotechnology.

[20] Schmidt M.S., Hubner J., Boisen A. (2012). Large area fabrication of leaning silicon nanopillars for surface enhanced Raman spectroscopy. Adv. Mater..

[21] Willets K.A., Van Duyne R.P. (2007). Localized Surface Plasmon Resonance Spectroscopy and Sensing. Annu. Rev. Phys. Chem..

[22] Fang Y., Seong N.-H., Dlott D.D. (2008). Measurement of the Distribution of Site Enhancements in Surface-Enhanced Raman Scattering. Science.

[23] Xu H., Aizpurua J., Käll M., Apell P. (2000). Electromagnetic contributions to single-molecule sensitivity in surface-enhanced Raman scattering. Phys. Rev. E.

[24] Xu H., Bjerneld E.J., Käll M., Börjesson L. (1999). Spectroscopy of Single Hemoglobin Molecules by Surface Enhanced Raman Scattering. Phys. Rev. Lett..

[25] Nie S., Emory S.R. (1997). Probing Single Molecules and Single Nanoparticles by Surface-Enhanced Raman Scattering. Science.

[26] Sau T.K., Murphy C. (2004). Room Temperature, High-Yield Synthesis of Multiple Shapes of Gold Nanoparticles in Aqueous Solution. J. Am. Chem. Soc..

[27] Vecchio G., Galeone A., Brunetti V., Maiorano G., Sabella S., Cingolani R., Pompa P.P. (2012). Concentration-Dependent, Size-Independent Toxicity of Citrate Capped AuNPs in Drosophila melanogaster. PLoS ONE.

[28] Zhang X.-D., Wu H.-Y., Wu D., Wang Y.-Y., Chang J.-H., Zhai Z.-B., Meng A.-M., Liu P.-X., Zhang L.-A., Fan F.-Y. (2010). Toxicologic effects of gold nanoparticles in vivo by different administration routes. Int. J. Nanomed..

[29] Zhu W., Banaee M.G., Wang D., Chu Y., Crozier K.B. (2011). Lithographically Fabricated Optical Antennas with Gaps Well Below 10 nm. Small.

[30] Mubeen S., Zhang S., Kim N., Lee S., Kraemer S., Xu H., Moskovits M. (2012). Plasmonic Properties of Gold Nanoparticles Separated from a Gold Mirror by an Ultrathin Oxide. Nano Lett..

[31] Hakonen A., Svedendahl M., Ogier R., Yang Z.-J., Lodewijks K., Verre R., Shegai T., Andersson P.O., Käll M. (2015). Dimer-on-mirror SERS substrates with attogram sensitivity fabricated by colloidal lithography. Nanoscale.

[32] Yue Z., Cai B., Wang L., Wang X., Gu M. (2016). Intrinsically core-shell plasmonic dielectric nanostructures with ultrahigh refractive index. Sci. Adv..

[33] Yue Z., Ren H., Wei S., Lin J., Gu M. (2018). Angular-momentum nanometrology in an ultrathin plasmonic topological insulator film. Nat. Commun..

[34] Lu H., Yue Z., Li Y., Zhang Y., Zhang M., Zeng W., Gan X., Mao D., Xiao F., Mei T. (2020). Magnetic plasmon resonances in nanostructured topological insulators for strongly enhanced light–MoS2 interactions. Light. Sci. Appl..

[35] Taflove A., Umashankar K.R. (1989). Review of FDTD numerical modeling of electromagnetic wave scattering and radar cross section. Proc. IEEE.

[36] Berenger J.P. (1994). A perfectly matched layer for the absorption of electromagnetic waves. J. Comput. Phys..

[37] Palik D. (1985). Handbook of Optical Constants of Solids.

[38] Rumble J. (1985). CRC Handbook of Chemistry and Physics.

[39] Le Ru E.C., Etchegoin P.G. (2006). Rigorous justification of the |E|4 enhancement factor in Surface Enhanced Raman Spectroscopy. Chem. Phys. Lett..

